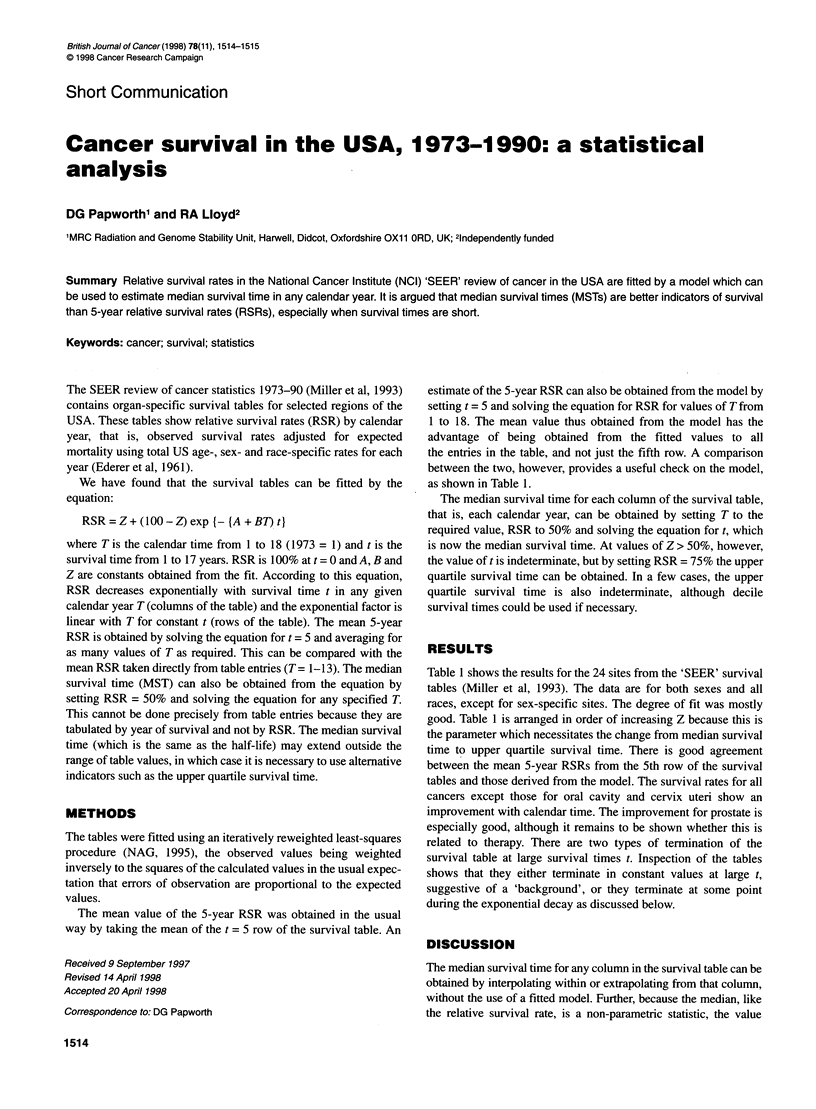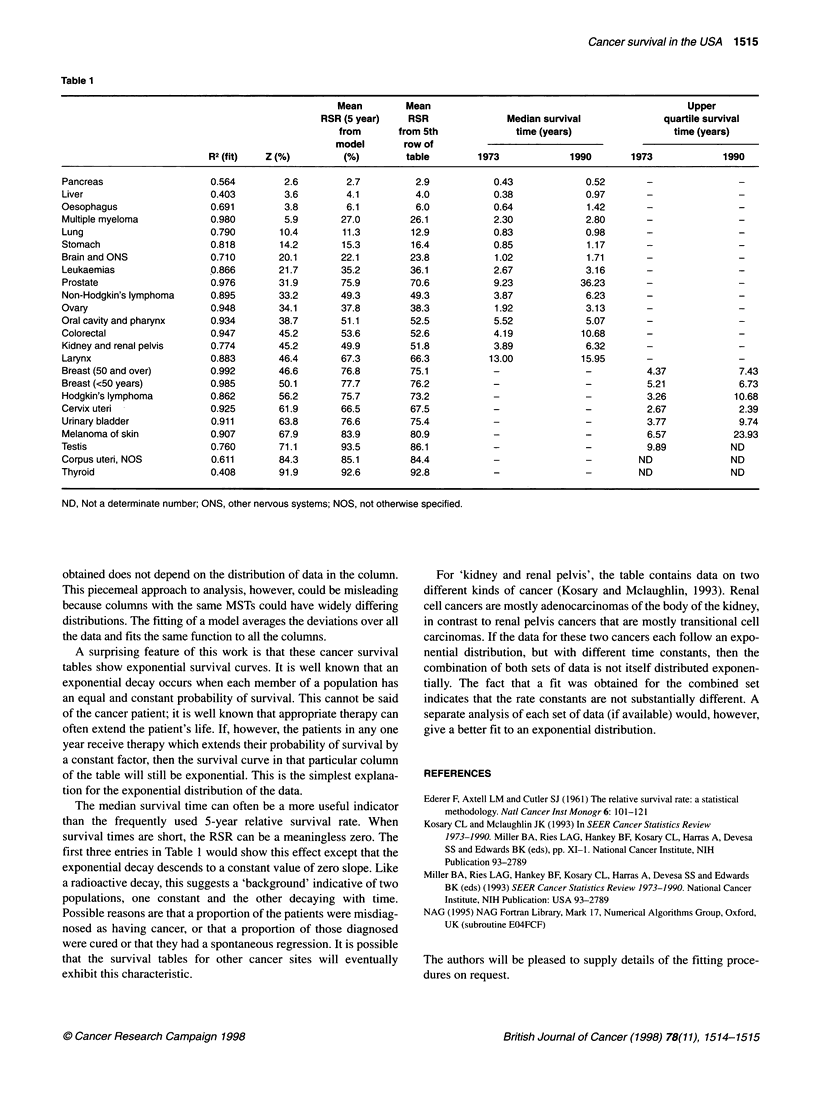# Cancer survival in the USA, 1973-1990: a statistical analysis.

**DOI:** 10.1038/bjc.1998.715

**Published:** 1998-12

**Authors:** D. G. Papworth, R. A. Lloyd

**Affiliations:** MRC Radiation and Genome Stability Unit, Didcot, Oxfordshire, UK.

## Abstract

Relative survival rates in the National Cancer Institute (NCI) 'SEER' review of cancer in the USA are fitted by a model which can be used to estimate median survival time in any calendar year. It is argued that median survival times (MSTs) are better indicators of survival than 5-year relative survival rates (RSRs), especially when survival times are short.


British Journal of Cancer (1998) 78(11), 1514–1515
© 1998 Cancer Research Campaign

Short Communication

### Cancer survival in the USA, 1973–1990: a statistical analysis

**DG Papworth[1] and RA Lloyd[2]**

[1]MRC Radiation and Genome Stability Unit, Harwell, Didcot, Oxfordshire OX11 0RD, UK; [2]Independently funded

**Summary** Relative survival rates in the National Cancer Institute (NCI) 'SEER' review of cancer in the USA are fitted by a model which can be used to estimate median survival time in any calendar year. It is argued that median survival times (MSTs) are better indicators of survival than 5-year relative survival rates (RSRs), especially when survival times are short.

**Keywords:** cancer; survival; statistics

The SEER review of cancer statistics 1973–90 (Miller et al, 1993) contains organ-specific survival tables for selected regions of the USA. These tables show relative survival rates (RSR) by calendar year, that is, observed survival rates adjusted for expected mortality using total US age-, sex- and race-specific rates for each year (Ederer et al, 1961).

We have found that the survival tables can be fitted by the equation:

$$RSR = Z + (100 - Z) \exp \{- \{A + BT\} t\}$$

where $T$ is the calendar time from 1 to 18 (1973 = 1) and $t$ is the survival time from 1 to 17 years. RSR is 100% at $t = 0$ and $A$, $B$ and $Z$ are constants obtained from the fit. According to this equation, RSR decreases exponentially with survival time $t$ in any given calendar year $T$ (columns of the table) and the exponential factor is linear with $T$ for constant $t$ (rows of the table). The mean 5-year RSR is obtained by solving the equation for $t = 5$ and averaging for as many values of $T$ as required. This can be compared with the mean RSR taken directly from table entries ($T$ = 1–13). The median survival time (MST) can also be obtained from the equation by setting RSR = 50% and solving the equation for any specified $T$. This cannot be done precisely from table entries because they are tabulated by year of survival and not by RSR. The median survival time (which is the same as the half-life) may extend outside the range of table values, in which case it is necessary to use alternative indicators such as the upper quartile survival time.

### METHODS

The tables were fitted using an iteratively reweighted least-squares procedure (NAG, 1995), the observed values being weighted inversely to the squares of the calculated values in the usual expectation that errors of observation are proportional to the expected values.

The mean value of the 5-year RSR was obtained in the usual way by taking the mean of the $t = 5$ row of the survival table. An estimate of the 5-year RSR can also be obtained from the model by setting $t = 5$ and solving the equation for RSR for values of $T$ from 1 to 18. The mean value thus obtained from the model has the advantage of being obtained from the fitted values to all the entries in the table, and not just the fifth row. A comparison between the two, however, provides a useful check on the model, as shown in Table 1.

The median survival time for each column of the survival table, that is, each calendar year, can be obtained by setting $T$ to the required value, RSR to 50% and solving the equation for $t$, which is now the median survival time. At values of $Z > 50\%$, however, the value of $t$ is indeterminate, but by setting RSR = 75% the upper quartile survival time can be obtained. In a few cases, the upper quartile survival time is also indeterminate, although decile survival times could be used if necessary.

### RESULTS

Table 1 shows the results for the 24 sites from the 'SEER' survival tables (Miller et al, 1993). The data are for both sexes and all races, except for sex-specific sites. The degree of fit was mostly good. Table 1 is arranged in order of increasing Z because this is the parameter which necessitates the change from median survival time to upper quartile survival time. There is good agreement between the mean 5-year RSRs from the 5th row of the survival tables and those derived from the model. The survival rates for all cancers except those for oral cavity and cervix uteri show an improvement with calendar time. The improvement for prostate is especially good, although it remains to be shown whether this is related to therapy. There are two types of termination of the survival table at large survival times $t$. Inspection of the tables shows that they either terminate in constant values at large $t$, suggestive of a 'background', or they terminate at some point during the exponential decay as discussed below.

### DISCUSSION

The median survival time for any column in the survival table can be obtained by interpolating within or extrapolating from that column, without the use of a fitted model. Further, because the median, like the relative survival rate, is a non-parametric statistic, the value

*Received 9 September 1997*
*Revised 14 April 1998*
*Accepted 20 April 1998*

*Correspondence to:* DG Papworth

**Table 1**

| | $R^2$ (fit) | Z (%) | Mean RSR (5 year) from model (%) | Mean RSR from 5th row of table | Median survival time (years) | | Upper quartile survival time (years) | |
|---|---|---|---|---|---|---|---|---|
| | | | | | 1973 | 1990 | 1973 | 1990 |
| Pancreas | 0.564 | 2.6 | 2.7 | 2.9 | 0.43 | 0.52 | – | – |
| Liver | 0.403 | 3.6 | 4.1 | 4.0 | 0.38 | 0.97 | – | – |
| Oesophagus | 0.691 | 3.8 | 6.1 | 6.0 | 0.64 | 1.42 | – | – |
| Multiple myeloma | 0.980 | 5.9 | 27.0 | 26.1 | 2.30 | 2.80 | – | – |
| Lung | 0.790 | 10.4 | 11.3 | 12.9 | 0.83 | 0.98 | – | – |
| Stomach | 0.818 | 14.2 | 15.3 | 16.4 | 0.85 | 1.17 | – | – |
| Brain and ONS | 0.710 | 20.1 | 22.1 | 23.8 | 1.02 | 1.71 | – | – |
| Leukaemias | 0.866 | 21.7 | 35.2 | 36.1 | 2.67 | 3.16 | – | – |
| Prostate | 0.976 | 31.9 | 75.9 | 70.6 | 9.23 | 36.23 | – | – |
| Non-Hodgkin's lymphoma | 0.895 | 33.2 | 49.3 | 49.3 | 3.87 | 6.23 | – | – |
| Ovary | 0.948 | 34.1 | 37.8 | 38.3 | 1.92 | 3.13 | – | – |
| Oral cavity and pharynx | 0.934 | 38.7 | 51.1 | 52.5 | 5.52 | 5.07 | – | – |
| Colorectal | 0.947 | 45.2 | 53.6 | 52.6 | 4.19 | 10.68 | – | – |
| Kidney and renal pelvis | 0.774 | 45.2 | 49.9 | 51.8 | 3.89 | 6.32 | – | – |
| Larynx | 0.883 | 46.4 | 67.3 | 66.3 | 13.00 | 15.95 | – | – |
| Breast (50 and over) | 0.992 | 46.6 | 76.8 | 75.1 | – | – | 4.37 | 7.43 |
| Breast (<50 years) | 0.985 | 50.1 | 77.7 | 76.2 | – | – | 5.21 | 6.73 |
| Hodgkin's lymphoma | 0.862 | 56.2 | 75.7 | 73.2 | – | – | 3.26 | 10.68 |
| Cervix uteri | 0.925 | 61.9 | 66.5 | 67.5 | – | – | 2.67 | 2.39 |
| Urinary bladder | 0.911 | 63.8 | 76.6 | 75.4 | – | – | 3.77 | 9.74 |
| Melanoma of skin | 0.907 | 67.9 | 83.9 | 80.9 | – | – | 6.57 | 23.93 |
| Testis | 0.760 | 71.1 | 93.5 | 86.1 | – | – | 9.89 | ND |
| Corpus uteri, NOS | 0.611 | 84.3 | 85.1 | 84.4 | – | – | ND | ND |
| Thyroid | 0.408 | 91.9 | 92.6 | 92.8 | – | – | ND | ND |

ND, Not a determinate number; ONS, other nervous systems; NOS, not otherwise specified.

obtained does not depend on the distribution of data in the column. This piecemeal approach to analysis, however, could be misleading because columns with the same MSTs could have widely differing distributions. The fitting of a model averages the deviations over all the data and fits the same function to all the columns.

A surprising feature of this work is that these cancer survival tables show exponential survival curves. It is well known that an exponential decay occurs when each member of a population has an equal and constant probability of survival. This cannot be said of the cancer patient; it is well known that appropriate therapy can often extend the patient's life. If, however, the patients in any one year receive therapy which extends their probability of survival by a constant factor, then the survival curve in that particular column of the table will still be exponential. This is the simplest explanation for the exponential distribution of the data.

The median survival time can often be a more useful indicator than the frequently used 5-year relative survival rate. When survival times are short, the RSR can be a meaningless zero. The first three entries in Table 1 would show this effect except that the exponential decay descends to a constant value of zero slope. Like a radioactive decay, this suggests a 'background' indicative of two populations, one constant and the other decaying with time. Possible reasons are that a proportion of the patients were misdiagnosed as having cancer, or that a proportion of those diagnosed were cured or that they had a spontaneous regression. It is possible that the survival tables for other cancer sites will eventually exhibit this characteristic.

For 'kidney and renal pelvis', the table contains data on two different kinds of cancer (Kosary and Mclaughlin, 1993). Renal cell cancers are mostly adenocarcinomas of the body of the kidney, in contrast to renal pelvis cancers that are mostly transitional cell carcinomas. If the data for these two cancers each follow an exponential distribution, but with different time constants, then the combination of both sets of data is not itself distributed exponentially. The fact that a fit was obtained for the combined set indicates that the rate constants are not substantially different. A separate analysis of each set of data (if available) would, however, give a better fit to an exponential distribution.

### REFERENCES

Ederer F, Axtell LM and Cutler SJ (1961) The relative survival rate: a statistical methodology. *Natl Cancer Inst Monogr* **6**: 101–121

Kosary CL and Mclaughlin JK (1993) In *SEER Cancer Statistics Review 1973–1990.* Miller BA, Ries LAG, Hankey BF, Kosary CL, Harras A, Devesa SS and Edwards BK (eds), pp. XI–1. National Cancer Institute, NIH Publication 93–2789

Miller BA, Ries LAG, Hankey BF, Kosary CL, Harras A, Devesa SS and Edwards BK (eds) (1993) *SEER Cancer Statistics Review 1973–1990.* National Cancer Institute, NIH Publication: USA 93–2789

NAG (1995) NAG Fortran Library, Mark 17, Numerical Algorithms Group, Oxford, UK (subroutine E04FCF)

The authors will be pleased to supply details of the fitting procedures on request.